# T Helper Cell Activation and Expansion Is Sensitive to Glutaminase Inhibition under Both Hypoxic and Normoxic Conditions

**DOI:** 10.1371/journal.pone.0160291

**Published:** 2016-07-28

**Authors:** Zeynep Sener, Fritjof H. Cederkvist, Roman Volchenkov, Halvor L. Holen, Bjørn S. Skålhegg

**Affiliations:** 1 Department of Nutrition, Institute of Basic Medical Sciences, University of Oslo, Oslo, Norway; 2 Rheumatech AS, 0855 Oslo, Norway; University of Iowa, UNITED STATES

## Abstract

Immune responses often take place where nutrients and O_2_ availability are limited. This has an impact on T cell metabolism and influences activation and effector functions. T cell proliferation and expansion are associated with increased consumption of glutamine which is needed in a number of metabolic pathways and regulate various physiological processes. The first step in endogenous glutamine metabolism is reversible and is regulated by glutaminase (GLS1 and GLS2) and glutamine synthase (GLUL). There are two isoforms of GLS1, Kidney type glutaminase (KGA) and Glutaminase C (GAC). The aim of this study is to investigate the expression, localization and role of GLS1 and GLUL in naïve and activated human CD4^+^ T cells stimulated through the CD3 and CD28 receptors under normoxia and hypoxia. In proliferating cells, GAC was upregulated and KGA was downregulated, and both enzymes were located to the mitochondria irrespective of O_2_ levels. By contrast GLUL is localized to the cytoplasm and was upregulated under hypoxia. Proliferation was dependent on glutamine consumption, as glutamine deprivation and GLS1 inhibition decreased proliferation and expression of CD25 and CD226, regardless of O_2_ availability. Again irrespective of O_2_, GLS1 inhibition decreased the proportion of CCR6 and CXCR3 expressing CD4^+^ T cells as well as cytokine production. We propose that systemic Th cell activation and expansion might be dependent on glutamine but not O_2_ availability.

## Introduction

As T cells reside in different lymphatic organs and bodily tissues, they must adapt to extensive environmental conditions including levels of nutrition availability and variable O_2_ tension. In tissues such as the lymph nodes and spleen O_2_ tension has been measured to be between 1-4kPa, whereas in the blood it is 6-13kPA (normoxia) and at sites of inflammation or tumor tissue it may be as low as ≤ 1kPA (hypoxia). It is well documented that the transcription factor hypoxia inducible factor 1-α (HIF-1α) is induced under hypoxic conditions and its expression leads to substantial metabolic changes in numerous cancer cells as well as T lymphocytes. However, even under normoxic conditions it has been shown that HIF-1α expression is augmented upon T cell activation stimulated through the T cell receptor (TCR)/CD3 complex and the CD28 receptor [1]. TCR/CD3 stimulation elicits a series of events eventually resulting in cell growth, proliferation, differentiation and production of a variety of cytokines [2]. During this process, T cells require energy in the form of ATP as well as electron donors such as nicotinamide adenine dinucleotide phosphate (NADPH) and large amounts of substrates for the production of biomass including lipids, proteins and DNA [3]. In order to supply for these metabolic demands, T cell metabolism is reorganized from mainly oxidative phosphorylation (oxphos) to aerobic glycolysis during which uptake of glucose through Glut1 is increased [4]. Pyruvate is a product of glycolysis which can be converted to lactate by lactate dehydrogenase [5]. This process, which is commonly called the Warburg effect, implies that the T cell mitochondria and the tricarboxylic acid (TCA) cycle are deprived of substrates coming from catabolizing glucose and indicates the need for alternative substrates to meet the demand for TCA intermediates and substrates for oxphos. It has been shown that T cell activation leads to an increased uptake of glutamine, facilitated by the glutamine transporter ASCT2 [6, 7]. Glutamine, which is the most abundant amino acid in circulation, is important for the biosynthesis and uptake of several amino acids for one-carbon metabolism and protein production, biosynthesis of pyrimidines and purines for RNA and DNA production, production of NADPH for macro molecule synthesis and finally synthesis of the anti-oxidant glutathione [8]. Carbon from glutamine can also contribute to the formation of citrate in the TCA cycle which when transported out of the mitochondrion into the cytoplasm contribute to the cytoplasmic Acetyl-Coenzyme A pool which may be used for lipid biosynthesis [9, 10]. Two pivotal enzymes regulate the initial reversible step in endogenous glutamine metabolism. The first is glutamine synthetase (GLUL), which synthesizes glutamine from glutamate and ammonia in an ATP-dependent reaction (EC 6.3.1.2, l-glutamine:ammonia ligase [ATP]). It has been demonstrated that GLUL plays a role in ammonia and glutamate detoxification, acid-base homeostasis and influences cell proliferation and keeps endogenous glutamine at a certain level [11, 12]. The second enzyme is glutaminase (GLS). GLS is the initial enzyme catabolizing glutamine to glutamate when glutamine enters the cell (EC 3.5.1.2, l-glutamine amidohydrolase). There are two isoforms of human GLS which are called GLS1 and GLS2. GLS1 exists as two splice variants called KGA and GAC. Whereas KGA is ubiquitously expressed, GAC is more specific to highly proliferating cell such as cancer cells [13, 14]. It has been shown that GLS activity increases upon T cell activation stimulated by α-CD3 and α-CD28 antibodies [15].

Bis-2-(5-phenylacetamido-1,2,4-thiadiazol-2-yl) ethyl sulfide, also known as BPTES, and compound 968 are well characterized inhibitors of GLS1 enzyme activity both *in vitro* and *in vivo* [16, 17]. They are both characterized as non-competitive inhibitors and bind to and function through distinct mechanisms to inhibit GLS1 [18, 19]. Crystal structure studies show that BPTES inhibits GLS1 in an allosteric fashion, whereas 968 inhibits GLS1 activity by preventing the enzyme to form tetrameric structures, and hence acts prior to enzyme activation [20]. Both inhibitors have been reported to have antitumor activity and BPTES has been shown to inhibit growth of a number of tumors [10, 21–24].

Glutamine dependence and the effect of O_2_ availability on cancer cell growth and development have been well documented [24–29]. However, comparably very few studies have revealed the effects of glutamine and various O_2_ levels on T cell proliferation, activation and cytokine production. Here we show that CD4^+^ T cells stimulated through CD3 and CD28 receptors, both under normoxic and hypoxic conditions, differentially regulate the levels but not localization of GLS1 and GLUL. We also show that BPTES and 968 inhibit α-CD3/CD28 induced CD4^+^ proliferation concomitantly with a decreased expression of the T cell activation markers CD25 and CD226. BPTES and 968 also differentially regulate cytokine production and proportion of CCR6 and CXCR3 expressing CD4^+^ T cells.

## Materials and Methods

### T cell isolation, culture and stimulation

Human CD4^+^ T cells were isolated from buffy coats of healthy donors (supplied by Blodbanken, Oslo, Norway) using the Dynabeads CD4 Positive Isolation kit (Life Technologies AS, Norway). Buffy coats were diluted with RPMI 1640 (1:2) and 1ml EDTA and rotated for 15min at 4°C. The kit was used to acquire CD4^+^ cells according to the supplied protocol. Five million cells/ml were maintained in RPMI 1640 (Sigma Aldrich, St. Louis, MO, USA) supplemented with 10% heat inactivated FBS (Sigma Aldrich); 2mM glutamine (Sigma Aldrich) 0.5% Penicillin-Streptomycin (Sigma Aldrich) unless stated otherwise. CD4^+^ T stimulation was initiated by adding washed Human T-Activator CD3/CD28 Dynabeads at a 1:4 bead/cells ratio (Life Technologies). CD4^+^ T cells were stimulated in this fashion during the entire study up to indicated time points.

### H^3^-Thymidine incorporation

Isolated CD4^+^ T cells were stimulated and cultured in DMEM 1X (no glucose, no glutamine) with 5% NaHCO3, 10% dialyzed FBS, 14 mM D-glucose and indicated amounts of L-Glutamine. Ten thousand cells were incubated in 100 μl of media under hypoxia (O_2_ = 1%) or normoxia (O_2_ = 20%) for 72 hours during which H3-thymidine (1μCi/well; PerkinElmer,Boston,MA,USA) was added to the media for the last 12 hours of incubation. H3-thymidine incorporation on the 72nd hour was obtained using a Wallac MicroBeta Counter (Perkin Elmer).

### Immunoblotting

Cell pellets were lysed by using lysis buffer containing 50mM Tris, 100mM NaCl,5mM EDTA, 50mM NaF, 50 mM Na_4_O_7_P_2_, 0.5% Triton X100. Total protein was determined by using Pierce BCA protein assay (Thermo Scientific, Rockford,USA). Protein lysates were denatured by boiling in SDS-sample buffer for 5 min at 95C° and loaded (25 μg/lane) onto 10% or 12.5% SDS-PAGE gels for protein separation. Separated proteins were transferred to pre-hydrated PVDF membranes (Millipore, Darmstadt, Germany) using the Criterion Blotter (Biorad,Hercules, CA) in a transfer buffer(25mM Tris-HCl, 190mM Glycine, 10% Methanol) for 45 min at 60V. Membranes were blocked with 5% milk powder in PBS for 45 min at RT. Membranes were incubated with antibodies recognizing GAC (Proteintech, Manchester, UK), KGA (ProteinTech,), GLUL (Abcam, Cambridge, UK), HIF-1α (Abcam) and β-Actin (Sigma) for 1 hour at RT under gentle agitation. Immune reactive proteins were detected using HRP-conjugated α-mouse (MPBio, Irvine, CA) or α-rabbit (MPBio) secondary antibodies, Supersignal Westdura Extended Duration substrate (Thermo Scientific) and the Syngene G:BOX imaging system(Cambridge,UK). Membrane stripping was done by incubating membranes with Restore Western Blot Stripping Buffer (Thermo Fisher) for 10 minutes at RT under gentle agitation.

### RT-QPCR

Total RNA was isolated with RNeasy plus Mini Kit (QIAGEN, Hilden,Germany) according to the manufacturer’s manual and RNA concentration was measured using NanoDrop 2000c Spectrophotometer (Thermo Scientific). Complementary DNA was synthesized using the Quantiscript Reverse Transcription Kit (QIAGEN). GAC (Life Technologies), KGA (Life Technologies), GLUL (Life Technologies), HIF-1α (Life Technologies) and Beta 2 microglobuline (B2M) (Life Technologies) probes were mixed with Taqman gene Expression master mix (Life Technologies) and cDNA to perform Quantitative PCR with ABI-7900 HT Fast Real-Time PCR (Applied Biosystems,Foster City,CA,USA). Ct values were obtained using SDS 2.3 program (Applied Biosystems). All data were normalized to internal reference B2M and relative expression determined with respect to unstimulated samples (ΔΔct (RQ)) using RQ Manager 1.2.1.

### Flowcytometry

Cells were labelled with 5 μM CFSE (ThermoFisher) for 10 min at 37 C° in PBS and immediately washed with 5 volumes of ice cold RPMI 1640 medium supplemented with 10% FBS to stop the staining. Cells were incubated with different concentrations of BPTES and 968 solubilized in DMSO, resulting in a final DMSO concentration of 0.33%. Samples were gated on live cells where dead cells were excluded using Violet live/dead cell stain (Life Technologies, L34955) for 15 minutes at RT. Proliferation index (PI) was calculated according to formula P.I. = $\sum_{0}^{i}Ni$/$\sum_{0}^{i}Ni$/2i where no proliferation gives an index value 1.

For T cell phenotype analysis, samples were washed with 2% FBS, 0.5 mM EDTA in PBS before cell surface staining with the following antibodies: CD25-APC (Biolegend, San Diego,CA,USA), CD183-PE (BD Biosciences, Heidelberg, Germany), CD196-PE-Cy7(BD Biosciences), CD161-FITC (Biolegend), CD45RO-PerCp-Cy5.5(BD Biosciences), CD45RA-BV605(BD Biosciences), CD226-PE(BD Biosciences). Live cells were gated from the forward and side scatter. For the mitochondrial biomass, Mitotracker Green FM (Thermofisher) was used in 100nM concentration and stained for 30 min. Data was acquired with the BD LSR Fortessa (Oslo University Flow Cytometry Core Facility) and analyzed using Flow-Jo V10 software (Tree Star, Ashland, OR).

### Metabolic Tracing

One million cells were incubated in the presence of 0.5 mM 15N2-13C5- Glutamine (Sigma Aldrich) and 5mM D-glucose (Sigma Aldrich) in DMEM no glucose,no glutamine medium and collected at 2 h by centrifugation (15000 rpm, 4°C). Metabolites of interest were extracted immediately by adding 300 μL 80% MeOH (-78°C) containing 10 μmol/L D5- Glutamine and D3- Glutamate as the internal standards. Cell debris was sedimented at 15000 rpm, 4°C for 15 min. Subsequently, supernatants were dried using a speed vac (DNA100 Speed Vac, ThermoTM SavantTM, USA) and stored at -78°C before being analyzed. For liquid-chromatography-tandem mass spectrometry (LC/MS-MS) analysis the samples were diluted in a mobile phase of 0.5% Formic acid (FA), 0.3% Heptafluorobutyric acid (HFBA). The labelled 15N2-13C5- glutamine and 15N-13C5- glutamate were separated isocratically by retention using a pheomenex kinetex core shell C18 (100 x 4.6 mm, 2.6 μm) column. The amounts of 15N2-13C5- glutamine were determined relative to the internal standard by integrating the area of each chromatographic peak. The LC-MS/MS analyses were performed on an Agilent 1100HPLC interfaced with a 4000 QTRAP linear MS/MS spectrometer (Applied Biosystems). The MS instrument was tuned for optimal conditions as follows: nebulizer gas 60 psi, electrospray voltage 3500 V, curtain gas 10 psi, temperature 650°C, GS1 50 psi, GS2 50 psi. The analytes were identified in positive ion mode (mw+1) and monitored in single ion mode using the optimized conditions for glutamine and glutamate, respectively: 15N2-13C5- Glutamine (mass 154/ 89, declustering potential 38 V, entrance potential 10 V, collision energy 18 V), 15N-13C5- Glutamate (mass 154/ 89, declustering potential 46 V, entrance potential 10 V, collision energy 24 V), D5IS- Glutamine (mass 152/89, declustering potential 38 V, entrance potential 10 V, collision energy 18 V), D3IS- Glutamate (mass 151/87, declustering potential 38 V, entrance potential 10 V, collision energy 18 V).

### Confocal microscopy

Glass covers were coated with Poly-D-Lysine and dried prior to use. Cells were resuspended in Krebs-Ringer buffer, incubated on a glass cover for 30 min at RT followed by rinsing with PBS. Cells were fixed using 4% formalin solution for 15 min, permeabilized with 0.01% Triton-X, blocked with 0.01% Tween, 3% BSA and 300mM glycine. Immunostaining was performed with 1:400 dilutions of primary antibodies: α-GAC (Proteintech), α-KGA (Proteintech), α-mitofilin (Abcam) and α-GLUL (Abcam,) for 1 hour at RT. Slides were subsequently incubated with Alexa-Flour 488 (Life Technologies, A-11008) and Alexa Flour 647 (Life Technologies, A-21235) conjugated secondary antibodies for 1h at RT in dark along with Hoechst (1:20000) nuclear counter stain. Glass cover slips were mounted with SlowFade Gold Antifade Mountant (Life Technologies) and sealed. Images were obtained using SuperAcochromat 60X objective(NA 1.35) with FluoView 1000 laser scanning confocal microscope (Olympus,Hamburg,Germany) and analyzed in ImageJ software (NIH,USA).

For the calculation of the Pearson Colocalization coefficient, we used the JACoP plug-in for Image J and visualized corresponding scatter plots. Values obtained from analysis vary from 1 to −1, with 1 indicating a complete positive correlation and −1 for a negative correlation, and zero meaning no correlation. Mid-range coefficients (−0.5 to 0.5) do not specify for biological assumptions [30]. Therefore, values between 0.5–1 have been chosen as an indicator of colocalization.

### Luminex Screening Assay

Supernatants from T cell cultures were harvested after 3 and 5 days of stimulation and stored at -20 C°. Samples were diluted two fold and human magnetic luminex screening assay (R&D Systems, Minneapolis, MN, USA) for TNF-α, IL-6, IL-10, INF-g, IL-4, IL-17α, IL-2 was performed according to manufacturer’s instructions. Data were analyzed with Milliplex Analyst (Merck Millipore) and standard curves set according to manufacturer’s recommendations.

### Statistics

Statistical analysis has been performed using GraphPad Prism 6 (La Jolla, CA,USA). For Q-PCR analysis the ratio t-test was used, two-way ANOVA was used for proliferation assays and flow cytometry and P values for the cytokine data was calculated using the student t test.

## Results

### CD4^+^ T cell proliferation stimulated through the CD3/CD28 receptors requires glutamine

**Fig 1:**
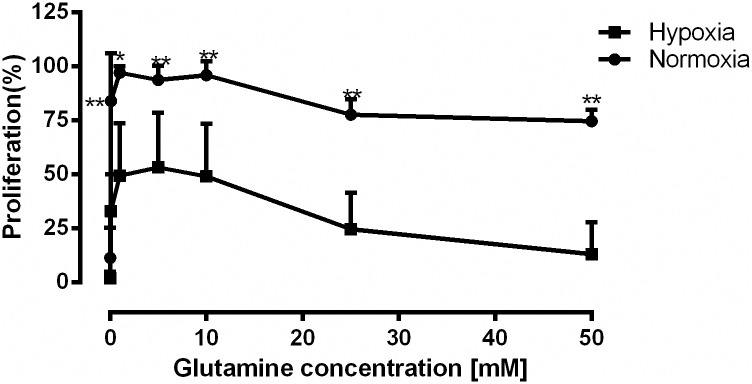
CD4^+^ T cell proliferation stimulated through the CD3 and CD28 receptors required glutamine. Human CD4^+^ T cells were stimulated through the CD3/CD28 receptors under either normoxic (O_2_ = 20%) or hypoxic (O_2_ = 1%) conditions in the absence and presence of incremental concentrations of glutamine (0.1–50 mM) for 72 hours. [3H] thymidine (40 μCi/ ml) was added for the last 24 hours of incubation. Maximal proliferation was measured under normoxic conditions at 1mM glutamine and it was set to 100%. Proliferation at all other concentrations was normalized accordingly. Proliferation was 50% slower under hypoxia and absence of glutamine attenuated cell proliferation. Data points represented proliferation of CD4^+^ T cells from three different donors (mean ± SD, ***p˂0.005, **p˂0.01, *p ˂0.05).

As metabolic requirements for the T cells might be different under different O_2_ concentrations, we determined the glutamine requirement for CD4^+^ T cells activated by α- CD3/CD28 coated beads under normoxic (O_2_≥20%) and hypoxic (O_2_ = 1%) conditions. Stimulation with incremental concentrations of glutamine (0–50 mM) for 72 h showed that proliferation under both normoxic and hypoxic conditions depended on the presence of glutamine and that the absence of glutamine completely abrogated proliferation (Fig 1). Proliferation reached its maximum at 1mM glutamine both under normoxia and hypoxia. At this concentration the proliferation rate was ~50% lower under hypoxia compared to normoxia.

### The expression of GAC, KGA and GLUL is differentially regulated under normoxia and hypoxia

**Fig 2:**
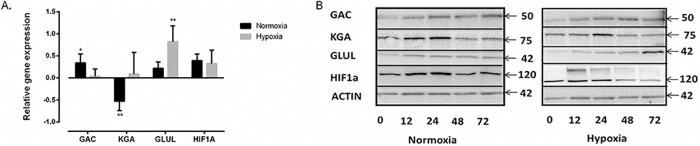
The expression of GAC, KGA and GLUL is differentially regulated under normoxia and hypoxia. (A) RT-qPCR analysis of *GAC*, *KGA*, *GLUL* and *HIF-1α* expression after 72 hours of α-CD3/CD28 stimulation. Gene expression values were presented normalized to unstimulated cells after 72h of incubation. *B2M* expression was used as an internal control and *HIF-1α* was used as a positive control. It showed significant upregulation of *GAC* and downregulation of *KGA* under normoxia and significant *GLUL* upregulation under hypoxia. Data were obtained from four independent experiments from three different donor samples each. (**p ˂0.01, *p˂0.05). (B) Western blot analysis of GAC, KGA, GLUL and HIF-1α after 0, 12, 24, 48 and 72 h of α-CD3/CD28 stimulated CD4^+^ T cells under normoxia and hypoxia. Under normoxia and hypoxia, GAC was upregulated and KGA was downregulated after a temporary increase. GLUL was upregulated only under hypoxia. HIF-1α was used as a control for hypoxic conditions and stimulation. β-Actin was used as a loading control and the images were obtained by stripping and re-probing the blots. The blots are representative examples of three independent experiments.

We next analyzed the expression levels of *GLS1* (*KGA* and *GAC*) and *GLUL*. Using specific primers for *KGA*, *GAC* and *GLUL*, we analyzed the mRNA levels by RT Q-PCR before and after 72 h of α-CD3/CD28 stimulation of CD4^+^ T cells under normoxia and hypoxia. In these experiments, relative mRNA expression was normalized to expression in unstimulated cells. After testing an internal standard library, *B2M* was chosen as an internal control since its mRNA levels did not significantly change under the stimulatory conditions used here. HIF1-α was shown to be upregulated upon stimulation both under normoxia and hypoxia. We showed (Fig 2A) that the *GAC* isoform was significantly upregulated upon stimulation (2.2 fold, p < 0.05) whereas *KGA* was downregulated (3.4 fold, p< 0.005) under normoxia. Whereas there were no significant differences in the expression levels of *GAC* and *KGA* under hypoxia, *GLUL* was significantly upregulated under this condition (6.62 fold, p< 0.005).

These results demonstrating differential regulation of *GAC*, *KGA* and *GLUL* at the mRNA level, encouraged us to investigate whether this also was reflected at the protein level. Results collected according to our western blot experiments and densitometric quantification (S1 Fig) showed that GAC protein levels were increased after 24h of stimulation both under normoxia and hypoxia (Fig 2B). By contrast KGA was downregulated in a biphasic fashion over a 72 h time course peaking at 24 h. GLUL levels were unaffected by the stimulatory conditions under normoxia but were significantly upregulated after 72 hours under hypoxic conditions. Together these data suggested for an isozyme switch of GLS1 from KGA to GAC upon stimulation through the CD3 and CD28 receptors under both normoxia and hypoxia. Moreover, the fact that GLUL levels were increased under hypoxic conditions may suggest for reversed glutamine metabolism under reduced O_2_ levels. To control for hypoxic as well as stimulatory conditions, we monitored the levels of immune reactive HIF-1α. This showed that HIF-1α expression was upregulated in a biphasic fashion during the course of stimulation both under normoxia and hypoxia. It should be noted that under hypoxia an immune reactive protein band of >120 kDa appeared 12h post stimulation. This protein has previously been shown to be a post translationally modified form of HIF-1α occurring under hypoxic conditions [31, 32].

### Relative localization of KGA, GAC and GLUL in quiescent and activated CD4^+^ T cells.

**Fig 3:**
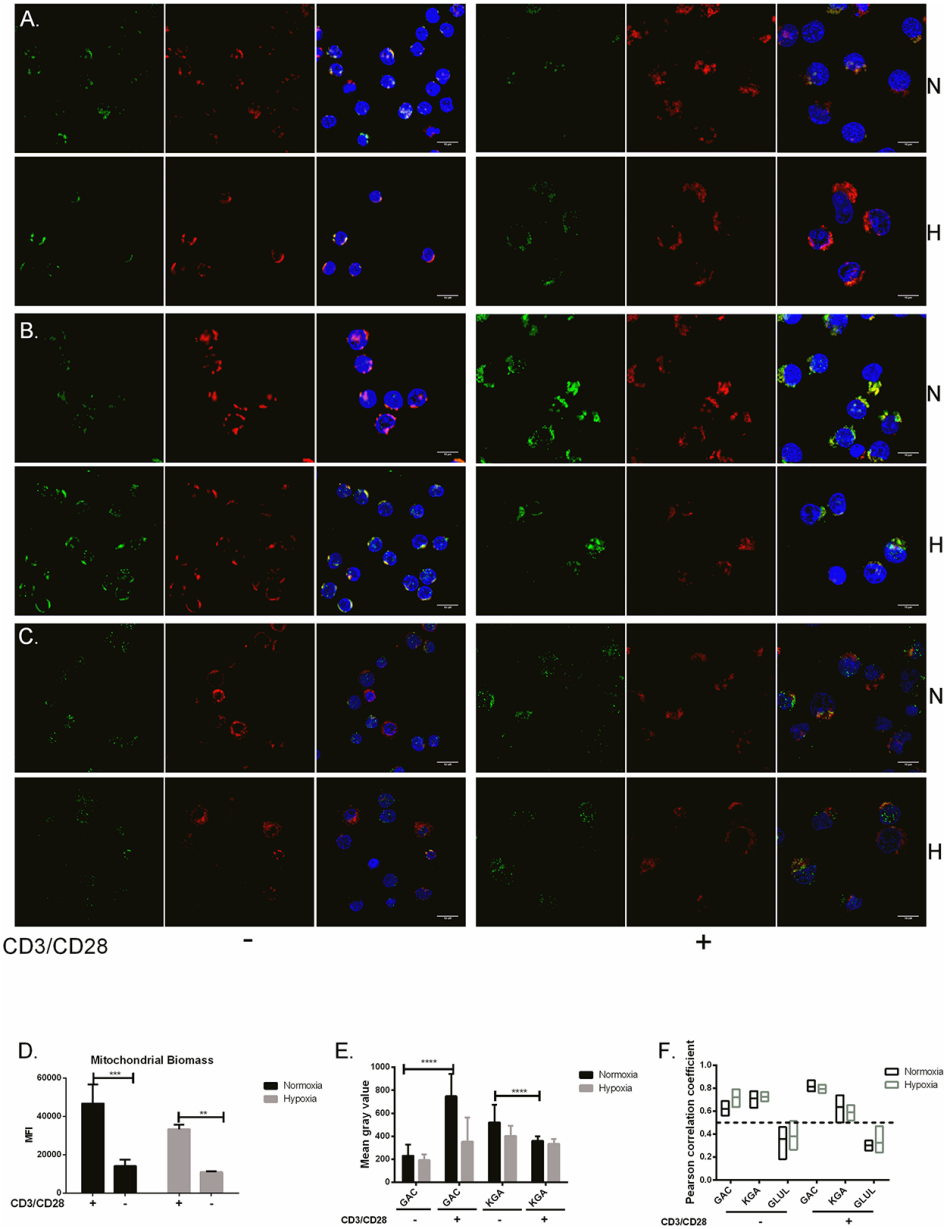
Localization of KGA, GAC and GLUL relative to mitofilin in quiescent and activated CD4^+^ T cells. α-CD3/CD28 stimulated (+) and unstimulated (-) CD4^+^ T cells incubated under hypoxia (H) and normoxia (N) were examined by confocal microscopy after immunofluorescence staining using Alexa Flour 488 (green) conjugated α-KGA (A), α-GAC (B) and α-GLUL (C) and α-mitofilin conjugated with Alexa-Fluor-647 (red). Cell nuclei were stained using DAPI (blue) and images were merged. The images are representative examples of three different experiments and showed apparent co-localization of GAC and KGA with mitochondria (Bar 10 μm). (D) Mitochondrial biomass was determined using Mitotracker Green FM and flow cytometry. This showed that mitochondrial biomass was significantly increased upon stimulation both hypoxia and normoxia. (***p< 0.0001 **p< 0.005) (E) Mean grey values for cells stained with α-GAC and α-KGA were determined by using the Image J software to determine individual image pixel intensity. Data points (n = 30) were collected from three different experiment and showed significant upregulation of GAC and downregulation in KGA upon stimulation under normoxic conditions (****p< 0.0001). (F) The localization of GAC, KGA and GLUL relative to the mitochondria was determined by calculating the Pearson correlation coefficient (PCC). Perfect linear correlation was given as 1 and lower cut-off for colocalization was set to 0.5. GAC and KGA were both localized with the mitofilin (PCC>0.5) whereas GLUL was not (PCC˂0.5).

Next we used KGA-, GAC- and GLUL-specific antibodies to evaluate the localization of these proteins relative to the mitochondria. Mitochondrial localization was detected by an antibody to the mitochondrion-specific protein mitofilin [33]. KGA, GAC and GLUL antibodies were all conjugated with Alexa-Fluor-488 (green) whereas mitofilin was conjugated with Alexa-Fluor-647 (red). Nuclei were stained with DAPI (blue). Fig 3 (panels A-C) showed the localization of KGA, GAC and GLUL relative to the mitochondria in unstimulated (-) and stimulated (+) cells under normoxia (N) and hypoxia (H). These data suggested mitochondrial localization of GAC and KGA and cytoplasmic localization of GLUL. Due to the fact that quiescent T cells have a relatively small cytoplasm, we observed that mitochondrial staining was weaker in unstimulated cells. This encouraged us to determine mitochondrial biomass under the stimulatory conditions used here. Using flow cytometry and Mitotracker Green FM, we showed that mitochondrial biomass in unstimulated cells was significantly lower than in stimulated cells both under hypoxia and normoxia. We also detected cells under hypoxia have less mitochondria compared to normoxia when stimulated (Fig 3D). Using the immunofluorescence data, we also determined the relative expression of GAC, KGA and GLUL by calculating Mean grey values. This was done from three different biological replicates where 30 cells were examined in each group. The calculated values indicated a significant (p> 0.0001) increase in GAC expression after stimulation under normoxia. Mean grey values for KGA were significantly (p< 0.0001) higher in unstimulated compared to stimulated cells under normoxia (Fig 3E) supporting our q-PCR and immunoblot results. We next quantified the localization of GAC, KGA and GLUL relative to mitofilin based on Pearson Correlation Coefficient (PCC) calculation of the images (Fig 3A–3C). In short, PCC quantitatively describes the colocalization of two variables where the cut-off for colocalization is set to > 0.5 and no colocalization is set to < 0.5. Based on this, we showed (Fig 3F) that KGA and GAC were localized to the mitochondria as determined by their relative localization to mitofilin both in unstimulated and stimulated cells (PCC > 0.5). GLUL, on the other hand, was consequently located to the cytoplasm (PCC < 0.5) under all conditions tested. Localization of GLUL, GAC and KGA were independent of hypoxic or normoxic conditions.

### The GLS1 inhibitors BPTES and 968 differentially regulate CD4^+^ T cell proliferation and activation under normoxia and hypoxia.

**Fig 4:**
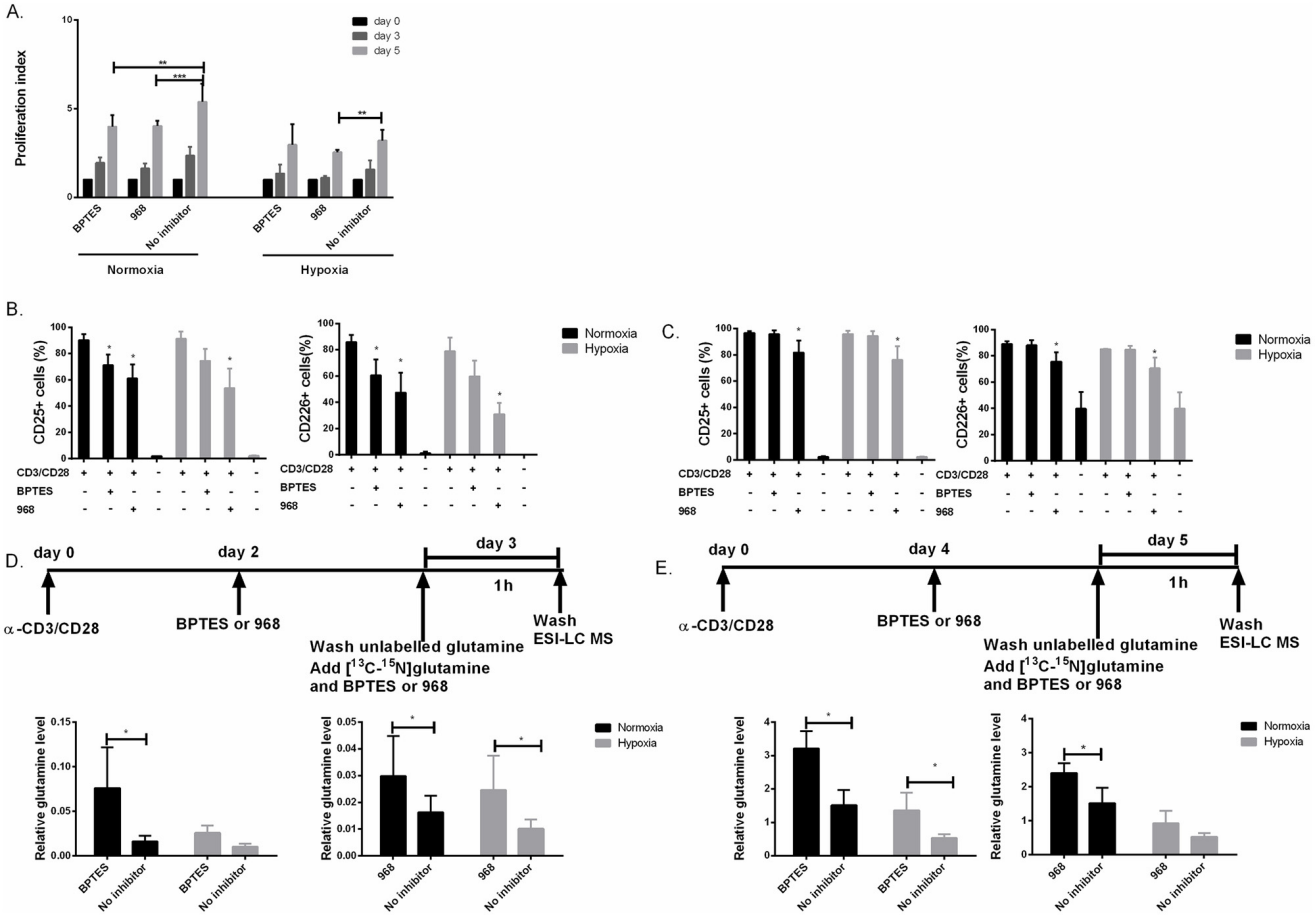
BPTES and 968 attenuated CD4^+^ T cell proliferation and activation under normoxia and hypoxia. (A) The proliferation index (PI) or average number of cell divisions, was calculated from CFSE dilutions of α-CD3/CD28 stimulated CD4^+^ T cells by flow cytometry at days 0, 3 and 5 in the absence (0 μM) and presence of (25 μM) BPTES and 968. Cell proliferation in 0.33% DMSO-containing media was used as control under normoxia and hypoxia. BPTES and 968 both reduced PI compared to control under normoxia. Under hypoxia, only 968 decreased the PI significantly (Mean ± SD, n = 4). (B) Expression of the cell surface activation markers CD226 and CD25 was lowered on day 3 by 968 and BPTES under both normoxic and hypoxic conditions. (C) On day 5, CD25 and CD226 expression was reduced by 968 but not significantly by BPTES both under normoxia and hypoxia. (D and E) Endogenous levels of [13C5-15N2]- glutamine was measured by ESI-LC MS after 3 (D) and 5 days of stimulation (E). [13C5-15N2] -glutamine was significantly increased in the presence of BPTES and 968 under normoxia both on day 3 and day 5. Under hypoxic conditions, only BPTES showed significant increase of intracellular glutamine on day 5. Data were normalized according to internal control (see material and methods). Data presented results of three separate experiments (n = 3) with 5 parallels in each experiments. (****p˂0.001, ***p˂0.005, **p ˂0.01, *p˂0.05)

To further assess the effect of glutamine on proliferating T cells under normoxic and hypoxic conditions, CD4^+^ T cells were stimulated in the absence and presence of the GLS1 inhibitors, BPTES and 968. Prior to these experiments dose response assays were performed to determine the potency (IC_50_) of BPTES and 968 to inhibit anti-CD3/CD28 induced CD4^+^ T cell proliferation. IC_50_ for BPTES and 968 were determined from experiments using more than 3 donors and results from two individual donors are depicted here (S2A Fig). The obtained data showed that IC_50_ values were between 25- and 35μM and 40- and 80 μM for BPTES and 968, respectively (S2A Fig). Based on these results, we used BPTES and 968 at doses between 25 and 50 μM in the following experiments as indicated. We next determined the proliferation index (PI) of T cells stimulated under normoxic and hypoxic conditions, in the absence (0 μM) and presence (25 μM) of BPTES and 968. PI was calculated by dividing the number of original parent cells (day 0) on the cell numbers on days 3 and 5, respectively (Fig 4A). In the presence of both BPTES and 968, PI was significantly reduced (p<0.05 and p<0.001, respectively) confirming the results from Fig 1, further confirming that T cell proliferation is dependent on the consumption and metabolism of glutamine. It should be noted that this was the case for both BPTES and 968 only under normoxia as PI appeared unaltered when employing BPTES under hypoxia. Only 968 was still able to inhibit proliferation under hypoxia, implying differential effects of these inhibitors on T cell proliferation. To control for non-physiological effects of the inhibitors, we also tested cell for viability and apoptosis by flow cytometry using propidium iodide and Annexin V staining (S2B Fig). This revealed that the cells were viable at concentrations used in the experiments. We also tested viability of the cells under hypoxia by violet live/dead staining as it has been reported that hypoxia might affect T cell survival and viability. We did not detect difference in viability between hypoxia and normoxia (S3 Fig).

**Fig 5:**
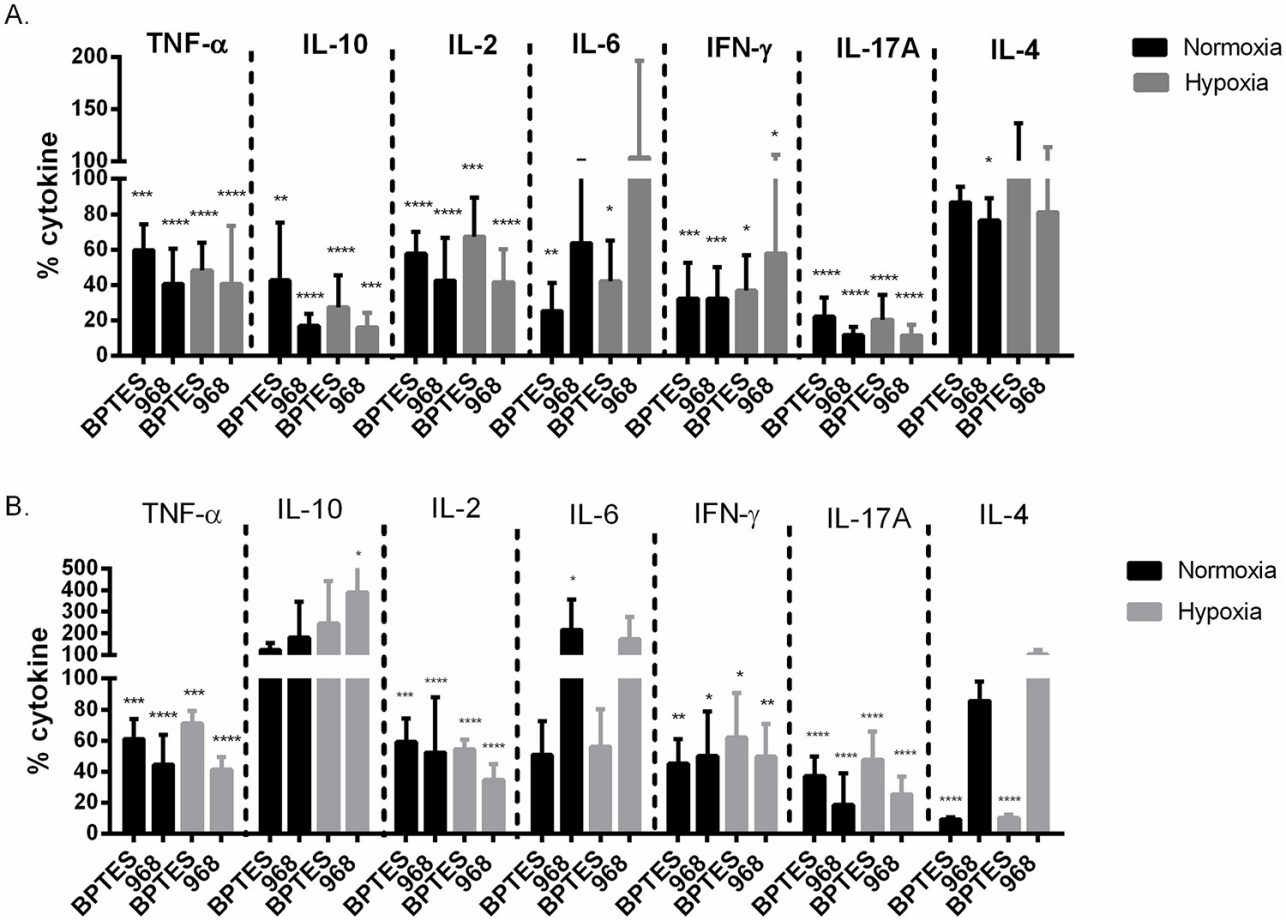
Inhibition of glutaminase attenuated cytokine secretion by activated CD4^+^ T cells. Levels of TNF-α, IL-10, IL-2, IL-6, INF-γ, IL-17A and IL-4 were measured in cell culture supernatants on day 3 (A) and 5 (B) of α-CD3/CD28 stimulated CD4^+^ T cells in the absence (only DMSO) and the presence of either 25 μM BPTES or 50 μM 968 under normoxic or hypoxic conditions, respectively. Cytokine levels were normalized to the corresponding stimulated sample containing DMSO. (A) BPTES reduced secretion of all cytokines measured except for IL-4 and 968 reduced all measured cytokines except for IL-6 both under normoxia and hypoxia on day 3. (B) BPTES lowered TNF- α, IL-2, IFN- γ, IL-17A and IL-4 secretion under both normoxia and hypoxia. 968 lowered TNF- α, IL-2, IFN- γ and IL-17A under normoxia and hypoxia. IL-10 secretion was significantly increased by 968 under hypoxia and IL-6 was increased under normoxia. Data presented were results of two independent experiments with three different biological replicates each. (Mean ± SD, ****p˂0.001, ***p˂0.005, **p˂0.01, *p ˂0.05).

The fact that T cell proliferation was significantly lower under hypoxia compared to normoxia and that inhibition of glutamine consumption and metabolism attenuated cell proliferation, encouraged us to monitor to what extent this was reflected by the expression of T cell activation markers such as CD69, CD25 (IL2Rα) and CD226 (DNAM-1). CD226 has been reported to be upregulated upon T cell stimulation, and contribute to activation by interacting with the CD155/CD112 molecules [34–36]. CD69 was not affected by the BPTES or 968 treatments at the 12^th^ and 18^th^ hours of stimulation (data not shown). CD25 and CD226 expressions were both increased by CD3/CD28 stimulation on day 3 (Fig 4B) and 5 (Fig 4C), and significantly reduced on day 3 (Fig 4B) in the presence of inhibitors BPTES and 968. It should be noted, on day 5, only 968 was able to significantly reduce the expression of both markers (Fig 4C).

Our results suggested that BPTES and 968 act differentially on T cell proliferation and expression of CD25 and CD226. Moreover, the fact that BPTES was less efficient in inhibiting cell proliferation and activation under hypoxia may suggest that BPTES was less effective in inhibiting glutamine metabolism or acted differently on this process at low O_2_ levels. To investigate this closer, we measured the intracellular conversion of [13C5-15N2]-glutamine to glutamate in the presence and absence of BPTES (50 μM) and 968 (75 μM) using ESI-LC MS. The concentrations of inhibitors were increased in these experiments to ensure rapid effects as they were added at the final step of incubation just 2 hours prior to harvesting for MS analysis (Fig 4D and 4E). We observed that endogenous glutamine levels were significantly increased in the presence of both BPTES and 968 under normoxia (p<0.05). However, only 968 had an effect on endogenous glutamine accumulation under hypoxia (p<0.05) on day 3 (Fig 4D). On the other hand, only BPTES led to endogenous glutamine accumulation both under normoxia and hypoxia on day 5 (Fig 4E).

### Inhibition of glutaminase attenuates cytokine secretion of activated CD4^+^ T cells.

To investigate if inhibition of glutamine metabolism and the presence of O_2_ influenced CD4^+^ T helper cell function, we measured the effects of BPTES and 968 on the key Th1 cytokines IL-2 and IFN-γ, Th2 cytokines IL-10 and IL-4, Th17 cytokine IL-17 as well as the pro-inflammatory cytokines TNF-α, and IL-6 on day 3 (Fig 5A) and day 5 (Fig 5B), both under normoxic and hypoxic conditions. BPTES significantly reduced the levels of all cytokines measured except for IL-4 on day 3. This was also the case for 968 except for the level of IL-6 which was not affected by this inhibitor (Fig 5A). On day 5, BPTES treatment reduced TNF- α, IL-2, IFN- γ and IL-17A and IL-4 secretion both under normoxia and hypoxia whereas 968 lowered TNF- α, IL-2, IFN- γ and IL-17A, also under normoxia and hypoxia (Fig 5B). Together these experiments imply that both BPTES and 968 decreased the secretion of all Th1 and Th17 cytokines under normoxia and hypoxia.

### Inhibition of GLS1 decreases the proportion of CCR6 and CXCR3 expressing CD4^+^ T cells.

**Fig 6:**
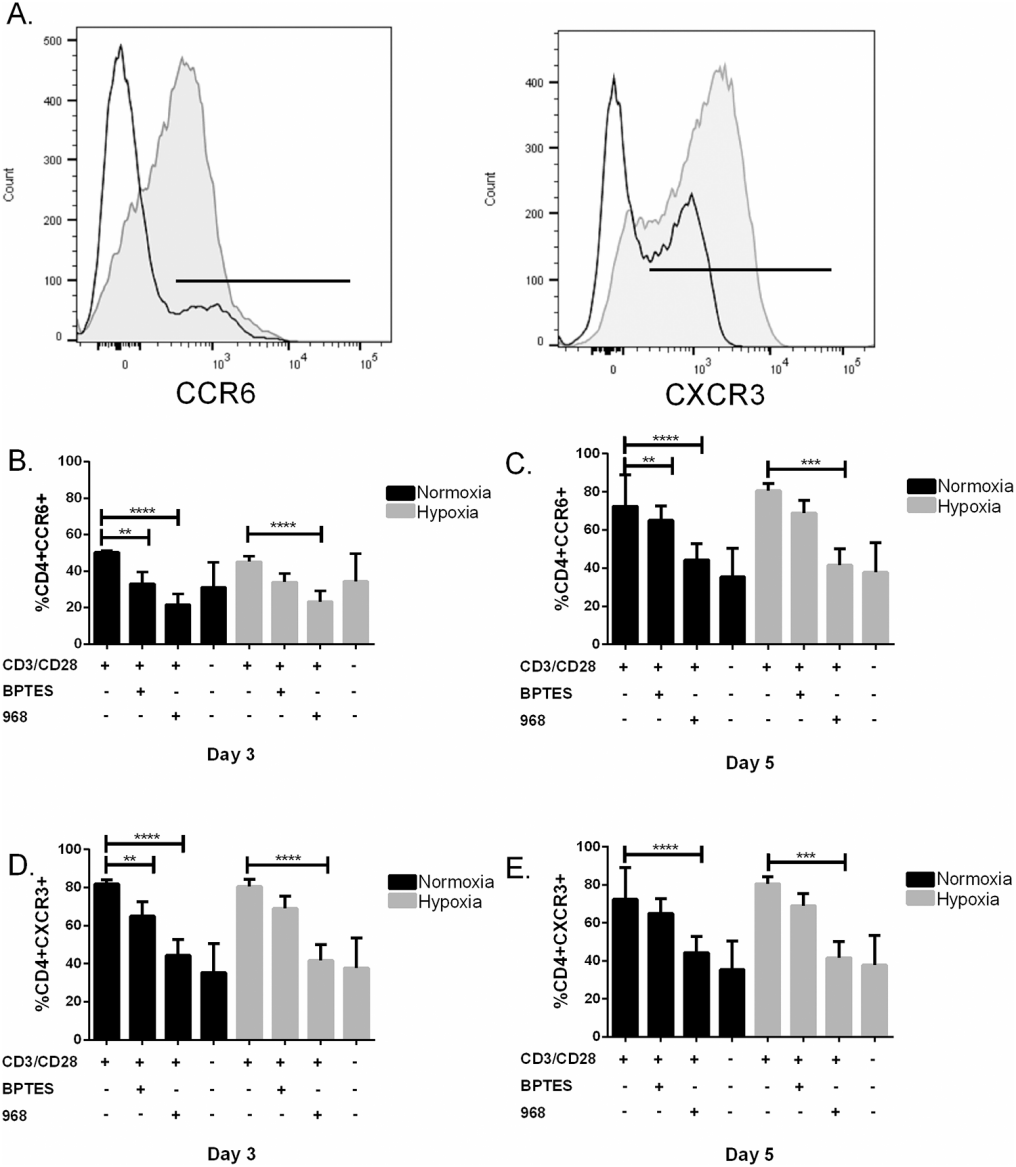
Inhibition of GLS1 decreases the proportion of CCR6 and CXCR3 expressing CD4^+^ T cells. (A) Representative flow cytometric analysis of the proportion of CCR6 (left) and CXCR3 (right) expressing CD4^+^ T cells stimulated with (grey) or without (open) anti-CD3/CD28 for 72 hours. (B–C) The proportion of CCR6 expressing cells at day 3 (left) and day 5 (right) under normoxia (black bars) and hypoxia (grey bars) in the presence (+) and absence (-) of 25 μM BPTES and 50 μM 968. BPTES and 968 significantly decrease the proportion of CCR6 expressing cells under normoxia both at day 3 and 5, whereas 968 but not BPTES decrease the proportion of CCR6 expressing cells under hypoxia both at day 3 and 5. (D–E) Expression of CXCR3 at day 3 (left) and day 5 (right) under normoxia (black bars) and hypoxia (grey bars) in the presence (+) and absence (-) of 25 μM BPTES and 50 μM 968. Both BPTES and 968 decreased the proportion of CXCR3 expressing cells under hypoxia at day 3 whereas only 968 decreased the proportion of CXCR3 expressing cells at day 3 under normoxia. At day 5 only 968 was capable of reducing the proportion of CXCR3 expressing cells both under hypoxia and normoxia. Data points in panels B-E represent results from three different donors (n = 3, Mean ± SD, ****p˂0.001, ***p˂0.005, **p˂0.01)

The observed overall decrease in cytokines secreted by CD4^+^ T cells when inhibiting GLS1 by BPTES and 968 encouraged us to analyzed the percentages of CD4^+^ T cells that expressed the chemokine receptors CCR6 and CXCR3 under the same stimulatory conditions. CXCR3 has been shown to be expressed mostly on Th1 cells and memory cells and CCR6 has been shown to be expressed on effector and memory T cells, Th17 cells and to some extend on Tregs [37–39]. This showed that BPTES (25 μM) and 968 (50 μM) significantly decreased the proportion of CCR6 expressing cells under normoxia both at day 3 and 5, whereas 968 but not BPTES reduced the proportion of CCR6 expressing cells under hypoxia both at day 3 and 5.(Fig 6B and 6C). Moreover, in the case of CXCR3 both BPTES (25 μM) and 968 (50 μM) decreased the proportion of CXCR3 expressing cells under hypoxia on day 3 whereas only 968 reduced the proportion of CXCR3 expressing cells on day 3 under normoxia. On day 5, only 968 was capable of reducing the proportion of CXCR3 expressing cells both under hypoxia and normoxia. Finally, no change in the proportion of cells expressing CD161, CD45RA and CD45RO were detected as a consequence of BPTES and 968 treatments (data not shown).

## Discussion

As described by others, immune reactions associated with T cell activation, proliferation, differentiation and clonal expansion requires nutrients such as glucose and glutamine for production of energy and biomass. Depending on the location in the body, such reactions will take place at different O_2_ partial pressures, which may influence the choice and flux of various metabolic pathways. The latter has been suggested to influence activity and fate of T cell sub populations. Here we show that expression of GAC, KGA and GLUL in CD4^+^ T cells are compartmentalized and differentially regulated. We also show that CD4^+^ T cell proliferation, activation, cytokine production as well as differentiation into T helper subtypes require functional glutamine metabolism and are independent of O_2_ levels.

We detected an upregulation of *GAC* and downregulation of *KGA* both at the mRNA and protein levels, demonstrating an isozyme switch upon α-CD3/CD28 stimulation. We also observed that both GAC and KGA are colocalized with the mitochondria in both quiescent and activated cells regardless of normoxia and hypoxia. We therefore propose that both KGA and GAC are involved in mitochondrial glutamine combustion to support TCA cycle intermediates and supply molecules for oxphos regardless of O_2_ availability even when the proliferation is low. The isozyme switch from KGA to GAC upon T cell activation may be associated with increased demand for glutamine by proliferating, compared to quiescent T-cells. A report by Cassago et *al*. demonstrated that GAC exhibits higher enzymatic activity compared to KGA even at concentrations as low as 10 mM Pi and that GACs activity increases concomitantly with increased concentrations of Pi [40]. Therefore, an isozyme switch from KGA to GAC upon T cell activation is consistent with meeting increased demand for glutamine turnover in proliferating cells where the need for biomass and energy is increased. However, we also observed the isozyme switch under hypoxia when proliferation was lower. This may imply that glutamine requirement is still high even when proliferation is lower than in normoxia. We also observed that GLUL, which converts glutamate to glutamine, was upregulated under hypoxia and located to the cytoplasm in proliferating cells. The physiological relevance of GAC/KGA and GLUL compartmentalization and ratio is not fully understood. However, it has been shown under hypoxia, cells uses reductive glutamine metabolism rather than using glucose oxidation for fatty acid synthesis [41]. We therefore speculate that upregulated GLUL may provide sufficient levels of glutamine in the cytoplasm under low O_2_ to support lipogenesis.

In our experiments on proliferating CD4^+^ T cells under hypoxia and normoxia, we regulated endogenous GLS1 activity by using the structurally distinct GLS1 inhibitors BPTES and 968. BPTES and 968 both reduced T cell proliferation, although BPTES appeared not to be as efficient as 968 under hypoxia. When examining glutamine levels by MS, we observed that 968, but not BPTES, increased endogenous levels of glutamine under hypoxia on day 3. On the other hand, 968 was inefficient in inhibiting GLS1 on day 5 under hypoxia. It has been demonstrated that BPTES is capable of inactivating GLS1 without disrupting its tetrameric form [42]. On the other hand, molecular docking models have further shown that 968 binds to the N-terminal end of GAC where the polymerization site is located and is not capable of binding to phosphate-activated GLS1 [16]. This implies that 968 is inefficient in inhibiting preformed GLS1. This may further imply that 968 will only inhibit GLS1 activity when enzyme turnover is high and new GLS1 enzymes are produced *de novo*. Our data on proliferation as well as the accumulation of endogenous glutamine may be explained by these modes of action by BPTES and 968. Furthermore, endogenous glutamine accumulation measured by MS during which the inhibitors were added at 2 and 4 days post-stimulation and endogenous glutamine levels measured on days 3 and 5, respectively. It showed that 968, but not BPTES, was efficient in significantly inhibiting glutamine consumption under hypoxia on day 3. This may imply that the GLS1 isozyme switch between KGA and GAC was ongoing and incomplete, explaining the effects of 968. On the other hand, on day 5 where 968 was ineffective, BPTES showed inhibitory effects. This may be explained by the fact that GLS1 turnover was low and GLS1 already formed rendering 968 inefficient. These proposed models are further supported by the immunoblot results demonstrating that the GAC-KGA isozyme switch occurs between 48 and 72 hours post α-CD3/CD28 stimulation.

BPTES and 968 incubations also reduced expression of the activation markers CD25 and CD226, with a stronger inhibitory effect seen by 968 under hypoxia while having no effect on CD69. It has been shown glutamine removal did not affect CD69 expression and it might be due to it is role during the early activation stage [43]. CD25 is also a well described T cell activation marker [44], CD226 was recently described as a T cell activation marker responsible for mediating cellular adhesion to cells expressing CD112 and CD155. CD226/CD155/CD112-interactions form networks resembling that of the CD28/CTLA4/CD80/CD86 interactions and regulate T cell activation [45]. It has been demonstrated that CD155 expressing cells compete for binding to CD226 with cells expressing the inhibitory molecule T-cell immunoglobulin and ITIM domain (TIGIT), and that this competition regulates the expression of INF-γ and IL-10 [46]. This means that CD226 interaction with CD155 leads to INF-γ production by the CD226^+^ cells. If, however, CD226 interacts with TIGIT the IL-10 production by the CD226^+^ cell will be upregulated [47]. We observed that GLS1 inhibition did not only reduced CD226 expression, but also significantly reduced INF-γ and IL-10 production on day 3. On day 5 neither CD226 nor INF-γ or IL10 were significantly altered. Together this suggests that glutamine metabolism may be involved in regulating the level of CD226 expression, and that glutamine in this fashion is indirectly capable of regulating CD226-mediated INF-γ and IL-10 production. Moreover, IL-10 is the key cytokine produced by Tregs and it has been shown that restricting glutamine metabolism can enrich FoxP3 expressing T cells [48, 49]. Since CD25 levels were not decreased on day 5, and IL-10 levels were increased, it may be speculated that FoxP3 expressing T cell populations were expanded by GLS1 inhibition. We also showed a decrease in the proportion of CCR6 and CXCR3 expressing CD4^+^ T cells producing IL-17 and IFN-γ. Both CCR6 and CXCR3 expressing CD4^+^ T cells have been identified in the synovial fluid of rheumatoid arthritis patients as well as in tissue samples of patients with other autoimmune diseases, such as multiple sclerosis [39, 50, 51]. Taking this into consideration, our results may suggest that reducing the availability of glutamine to the CD4^+^ cells might have beneficial effect on reducing T cells promoting autoimmunity by decreasing pathogenic CCR6 and CXCR3 bearing T cell and increasing regulatory T cells.

To this date, only a limited number of studies have examined the metabolic demands for human CD4^+^ T cell activation and expansion [52, 53]. In line with previous studies, we found that hypoxic conditions were associated with a 50% reduction in proliferation without affecting cell viability. Additionally, we observed that CD25 and CD226 expression as well as the decrease in CCR6 and CXCR3populations were all events which occurred independently of hypoxic or normoxic conditions but might be dependent on glutamine. We speculate that the ratio and localization of GLS1 and GLUL are tightly controlled to maintain sufficient levels of compartmentalized glutamine to support optimal T cell proliferation and expansion, both under normoxic and hypoxic conditions. Physiologically, this may indicate that T helper cells may require glutamine and may occur in hypoxic tissues as long as nutrients such as glutamine are sufficiently supplied either through exogenously or by endogenous synthesis via GLUL.

## Supporting Information

S1 Fig Densitometric quantification of the represented western blot was calculated from sthe band intensities of GAC (A), KGA (B), GLUL (D) and HIF1-α(D) with respect to β-actin control on 0, 12 24,48, 72 hours. (TIF)

S2 Fig (A) Dose-dependent inhibition of anti-CD3/CD28 induced CD4^+^ T cell proliferation by incremental concentrations of BPTES (upper panels) and 968 (lower panels). CD4^+^ T cells from two individual donors were incubated with the inhibitors for 72 hours and showed IC_50_ values between 25- and 60 μM for BPTES and 40 and 80 μM for 968. (B) Annexin V and PI staining of anti-CD3/CD28 stimulated CD4^+^ T cells in the presence of BPTES and 968 after 72 hours incubation. The concentrations of the inhibitors used in the experiments are neither apoptotic nor necrotic to the cells.
(TIF)

S3 Fig Violet live/dead stain of cells under hypoxia (grey bars) and normoxia (black bars) shows that the viability of CD4^+^ T cells under hypoxia is not significantly different from viability of the CD4^+^ T cells under normoxia. (TIF)

S4 Fig CD4^+^ T cells cytokine levels in the absence (anti-CD3/CD28 stimulation) and presence of inhibitors (BPTES and 968) treated on day 3 and day 5 (A and B, respectively). (TIF)
