# Supplementary figures and images for: T Helper Cell Activation and Expansion Is Sensitive to Glutaminase Inhibition under Both Hypoxic and Normoxic Conditions
Source: PLoS One. 2016 Jul 28;11(7):e0160291. doi: 10.1371/journal.pone.0160291 (PMC4965213; doi:10.1371/journal.pone.0160291)

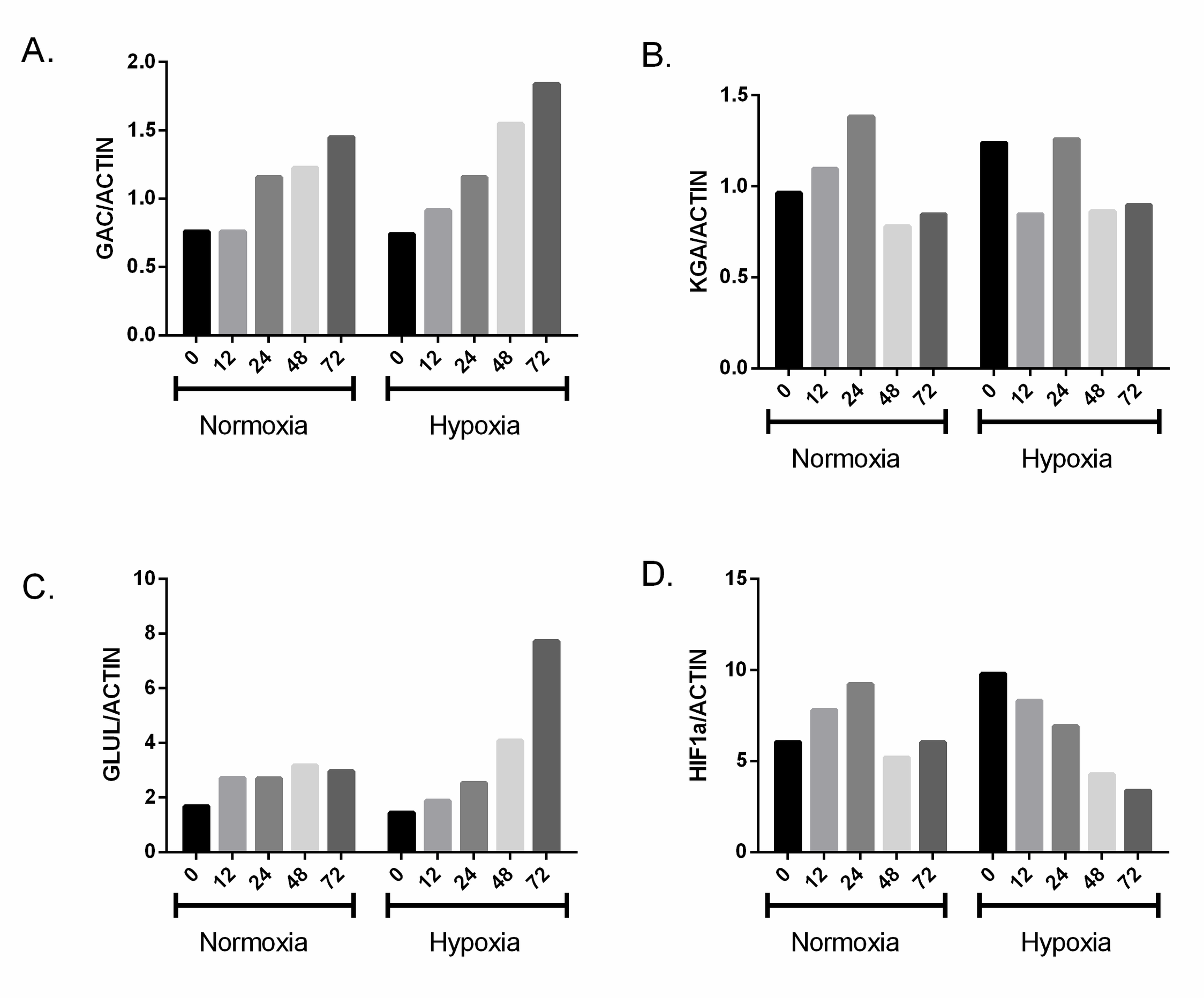

Supplement: S1 Fig — (TIF) [file pone.0160291.s001.tif]

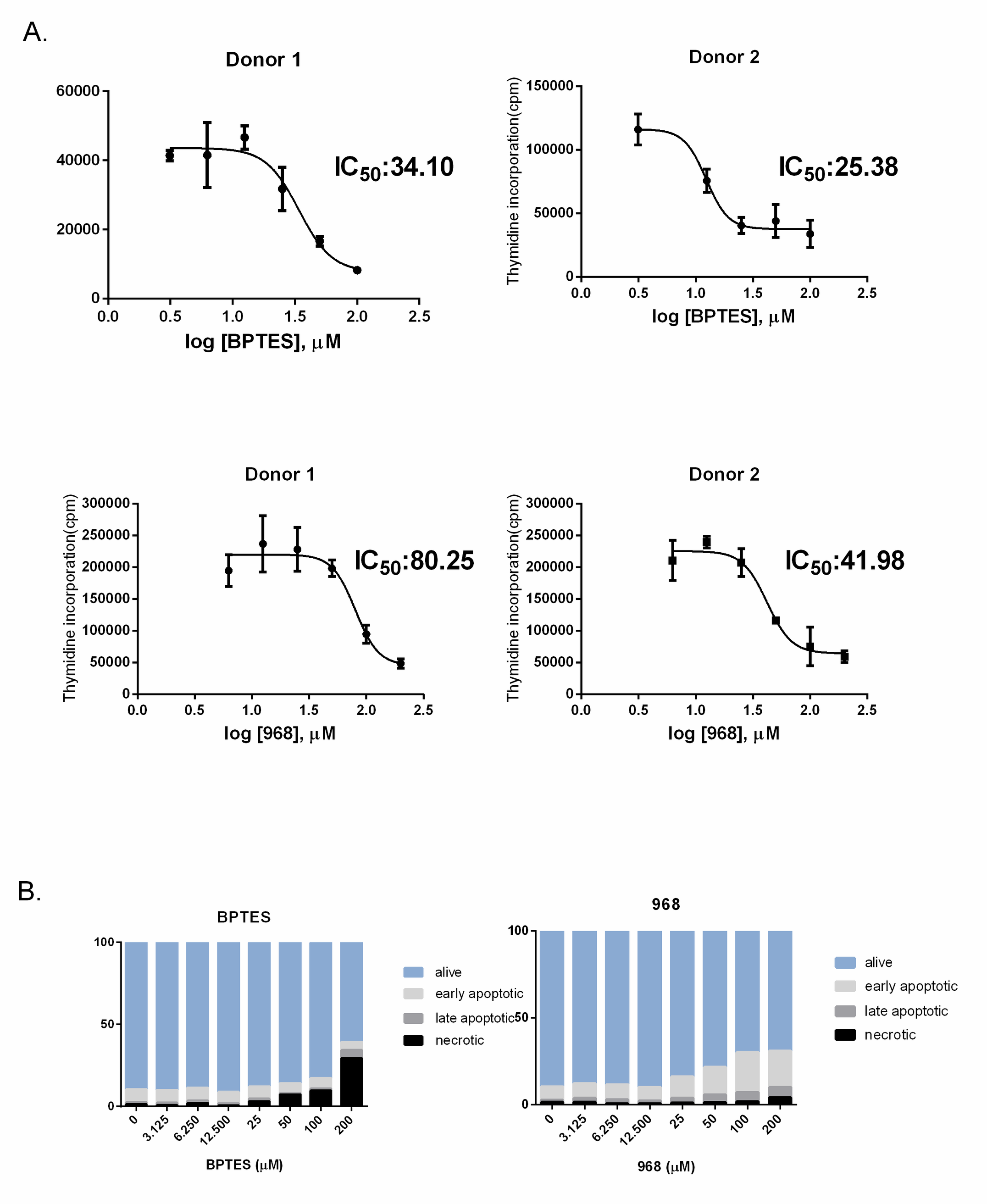

Supplement: S2 Fig — (A) Dose-dependent inhibition of anti-CD3/CD28 induced CD4+ T cell proliferation by incremental concentrations of BPTES (upper panels) and 968 (lower panels). CD4+ T cells from two individual donors were incubated with the inhibitors for 72 hours and showed IC50 values between 25- and 60 μM for BPTES and 40 and 80 μM for 968. (B) Annexin V and PI staining of anti-CD3/CD28 stimulated CD4+ T cells in the presence of BPTES and 968 after 72 hours incubation. The concentrations of the inhibitors used in the experiments are neither apoptotic nor necrotic to the cells. (TIF) [file pone.0160291.s002.tif]

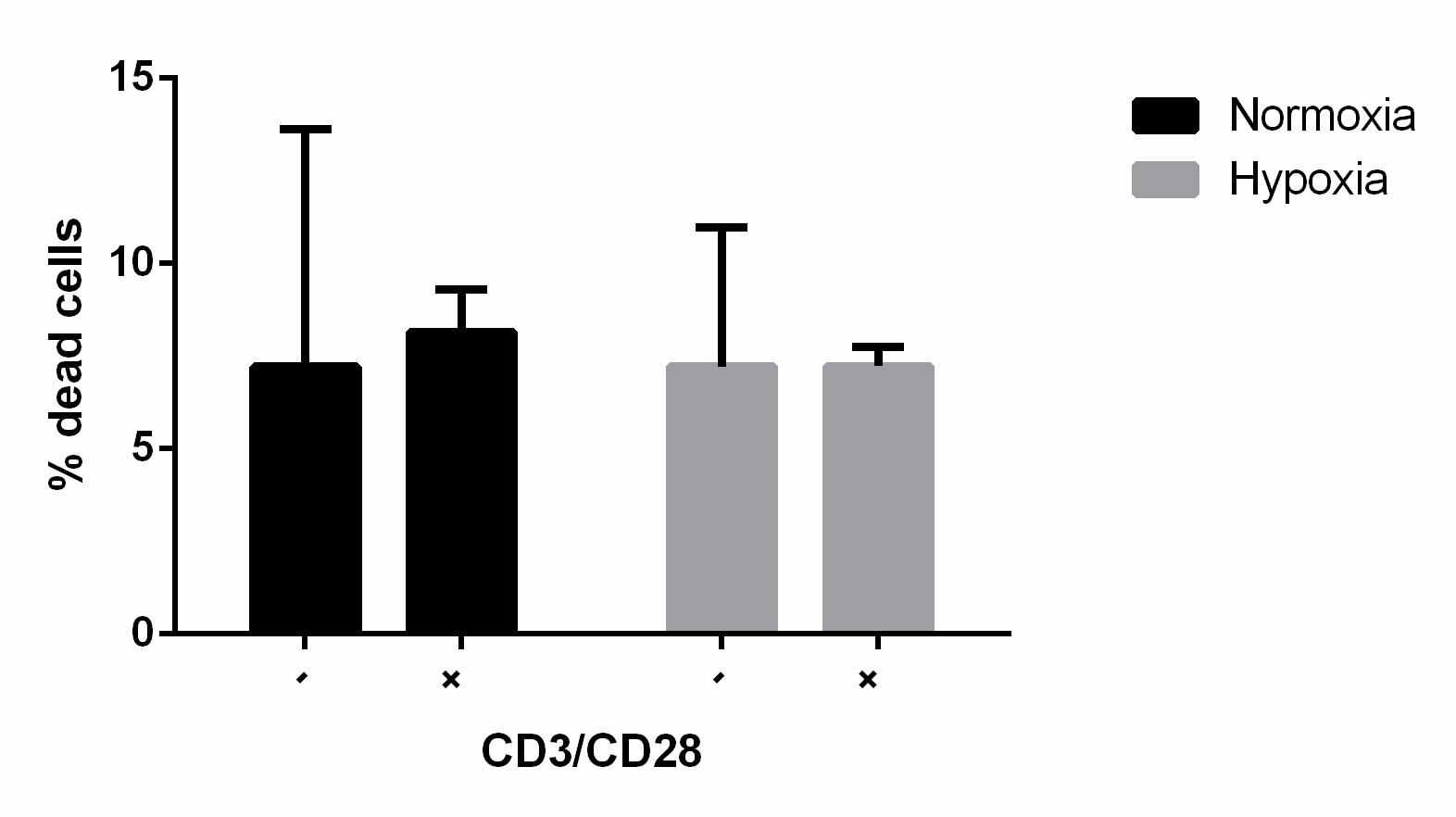

Supplement: S3 Fig — (TIF) [file pone.0160291.s003.tif]

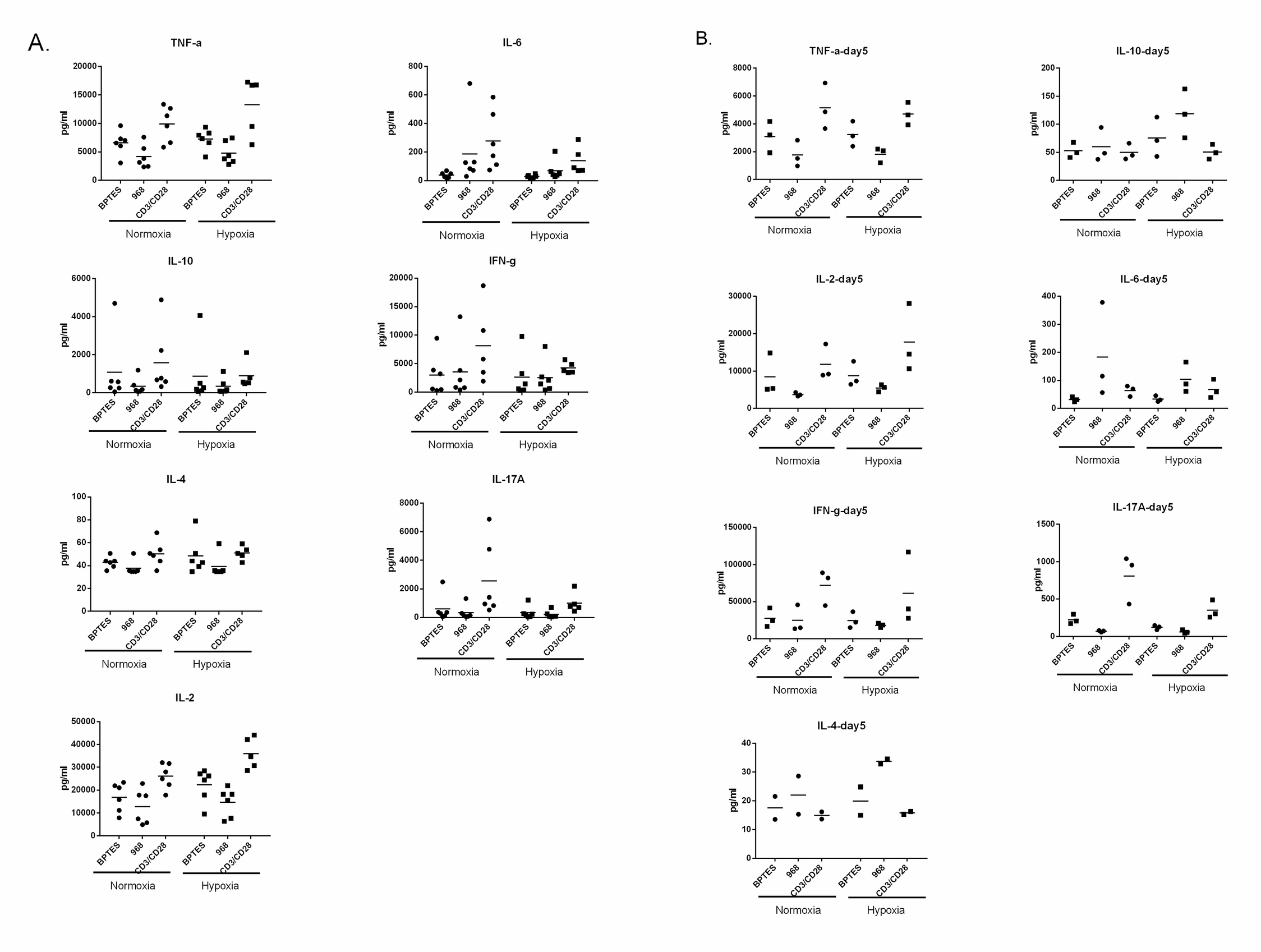

Supplement: S4 Fig — (TIF) [file pone.0160291.s004.tif]
